# Determination of Microplastic Pollution in Commercial Fish in the Middle Black Sea (Samsun), Türkiye

**DOI:** 10.3390/toxics13100865

**Published:** 2025-10-12

**Authors:** Arife Şimşek

**Affiliations:** Hemp Research Institute, Ondokuz Mayıs University, Samsun 55139, Türkiye; arife.simsek@omu.edu.tr

**Keywords:** aquaculture, Black Sea, commercially sold fish, microplastics

## Abstract

This study aimed to determine the presence and characteristics of microplastics (MPs) in six commercially important fish species in Samsun city of, the Middle Black Sea Region: rainbow trout–Turkish salmon (*Oncorhynchus mykiss*), European seabass (*Dicentrarchus labrax*), gilthead seabream (*Sparus aurata*), red mullet (*Mullus barbatus*), horse mackerel (*Trachurus mediterraneus*), and whiting (*Merlangius merlangus*). The digestive systems of each species were examined, and MPs were classified according to their morphology, size, color, and polymer type. The analysis revealed that the number of MPs per individual ranged from 4.73 ± 1.13 to 9.26 ± 2.18, with the highest value found in rainbow trout and the lowest in whiting. MPs smaller than 100 µm were dominant (48.9%), and fiber (45.7%) and fragment (36.5%) types were the most common morphologies observed. Black and white/transparent colors were prominent in terms of color distribution, and ATR-FTIR analysis showed a dominance of widely used consumer plastics, such as polypropylene (PP, 31.3%) and polyethylene (PE, 23.9%). Scanning electron microscopy coupled with energy-dispersive X-ray spectroscopy (SEM/EDS) results confirmed the presence of irregular, fibrous, and fragmented structures at microscopic scale, consistent with microplastic morphology. These findings indicate a potential risk of microplastic pollution in the region for both marine biota and human consumption. The study fills a significant data gap regarding the Middle Black Sea ecosystem and provides a foundation for future monitoring and risk assessment research.

## 1. Introduction

Plastics are widely used in various fields due to their high applicability and durability, with their production reaching 390 million tons in 2022 [1]. However, only a small portion of these plastics are recycled or reused. Large amounts of plastic debris enter aquatic environments as waste, leading to plastic pollution [2]. Microplastics (MPs) are generally defined as plastic particles smaller than 5 mm [3,4], and they enter marine environments primarily through river discharges [5,6], wastewater treatment plant effluents [7], surface runoff [8], and atmospheric deposition [9]. Due to their small size, low density, and buoyancy, MPs can easily be mistaken for food and ingested by marine organisms [10,11,12].

Studies to date have shown that MP ingestion is widespread among various marine organisms, including zooplankton [13,14,15], bivalves [16,17], crustaceans [16,18], and fish [19,20,21]. Ingestion of microplastics can lead to several adverse effects, including gastrointestinal blockage [22], absorption of nano- and micro-sized particles by intestinal and other tissues causing bioaccumulation [18,23,24], and the role of these particles as vectors for pollutants, facilitating the transfer of toxic chemicals to organisms [25,26]. Through the marine food web, MPs can affect not only marine organisms, but also humans who consume these organisms, posing a potential risk to human health [21,27,28].

As the number of MPs in aquatic environments increases, so do their interactions with aquatic fauna. Many aquatic organisms, such as plankton [13], aquatic plants [29,30], fish [21,31], and marine mammals [32], are adversely affected by MPs due to their small size and buoyant properties [10]. Fish, in particular, are reported to be highly vulnerable to MP ingestion due to the similarity of MPs to their prey, high buoyancy, and the variable size and shape of MPs [33,34]. The bioaccumulation, biomagnification [35], and transport [36] of MPs and associated pollutants within the food chain increase the potential exposure and health risks for consumers at higher trophic levels [35,37,38]. MP ingestion may also trigger immune responses, leading to tissue damage and chronic inflammation [39,40].

The ingestion of microplastics (MPs) by fish has become an emerging field of research that has received increasing attention [39,41]. Field studies confirm that both commercial and non-commercial fish species from different habitats and trophic levels ingest MPs, yet the full extent of chemical exposure and the implications for seafood safety remain unclear [22,42,43]. The evidence suggests that certain species, such as herbivores, may be more prone to MP ingestion than others, and marine fish may ingest food more rapidly than freshwater species [44]. Seafood consumption is therefore recognized as a pathway for human MP exposure, with studies reporting that a significant proportion of MPs in fish tissues can be transferred to humans [45]. This raises potential health risks, as suggested by recent findings on animals ingesting MPs [46,47], while experimental studies have shown that particles such as PS and PVC can reach the lymphatic and circulatory systems through ingestion [48].

In Türkiye, the aquaculture production volume has more than doubled in the past decade [49]. Rainbow trout, gilthead seabream, and European seabass are the most widely farmed fish species in the country, and the total aquaculture production increased by 18.6% in 2023 compared to the previous year [49]. As a result of this high production volume, Türkiye is one of the leading producers of these species in the Black Sea and Mediterranean regions [50]. Although some studies have investigated the presence of MPs in fish along Türkiye’s Southeastern and Western Black Sea coasts [37,51,52,53], no studies have been conducted on MP contamination in fish raised and consumed in the Middle Black Sea Region. While studies examining wild marine and freshwater species are increasing [54], studies focusing on MP intake in farmed fish remain significantly limited.

This study aims to determine the levels of MPs in highly consumed fish species that are either farmed or caught from the sea in the Mid-Black Sea Region of Türkiye. It also seeks to fill the knowledge gap regarding MP abundance and sources that pose a threat to both fish and human health. The primary objective is to quantify the number of MPs in the gastrointestinal systems (GIS) of fish samples, to characterize their morphological features (size and shape), to identify their chemical composition (polymer types and colors) via attenuated total reflectance (ATR) and Fourier transform infrared (FTIR) spectroscopy analysis, and to confirm the findings through scanning electron microscopy (SEM). By identifying potential variations in MP abundance across fish habitats, this study aims to contribute to public awareness and food safety, in line with the European Green Deal, by revealing the amount and types of MPs in fish intended for human consumption.

## 2. Materials and Methods

### 2.1. Sampling

For this study, six fish species caught and raised along the Mid-Black Sea were selected: *Oncorhynchus mykiss* (rainbow trout–Turkish salmon), *Dicentrarchus labrax* (European seabass), *Sparus aurata* (gilthead seabream), *Mullus barbatus* (red mullet), *Trachurus mediterraneus* (horse mackerel), and *Merlangius merlangus* (whiting). These species are among the most commonly caught and consumed along the Black Sea coastal regions of Türkiye [49]. While rainbow trout was raised in fresh water, gilthead seabream and European sea bass specimens were raised in marine environments. A total of 90 fish samples (*n* = 15 per species) were collected: *M. barbatus*, *T. mediterraneus*, and *M. merlangus* were obtained between 1 and 15 April 2025, due to the closure of the fishing season, while *O. mykiss*, *D. labrax*, and *S. aurata* were obtained from local fishers between 1 and 15 July 2025. All specimens examined were recently caught and showed no signs of morphological deformation.

Additionally, the selected fish species were chosen to represent three different zones of the water column: demersal, benthopelagic, and pelagic. General information on the examined species and sample numbers from the Mid-Black Sea Region (Samsun) is presented in Table 1. The fish samples were wrapped in aluminum foil, transported to the laboratory, and stored at −20 °C until extraction.

### 2.2. Microplastic Extraction

Prior to extraction, the weight and total length of each of the 90 fish samples (15 individuals per species) were measured and recorded. The MP extraction procedure was adapted from methods described by [40,52,55]. To prevent contamination during dissection, fish samples were rinsed with filtered ultrapure water before extracting the gastrointestinal tracts (GITs). A stainless-steel dissection kit (Microtest, Ankara, Türkiye) was used inside a laminar flow cabinet to remove the GITs of each specimen.

Each GIT was placed into separate 500 mL glass bottles. Subsequently, 30% hydrogen peroxide (H_2_O_2_, Tekkim, Bursa, Türkiye) was added at a ratio of 20 mL per gram of tissue [56], and the bottles were tightly sealed. The samples were incubated for 24 h at 65 °C in a temperature-controlled shaking incubator to ensure the complete digestion of organic matter.

Following digestion, the solutions were filtered using a vacuum glass filtration system with 1.2 µm pore size glass fiber filters (Glass Fiber Filter 47 mm–1.2 μm, Borox, İstanbul, Türkiye). The filters were then placed in glass Petri dishes and left to dry for subsequent microscopic analysis.

### 2.3. Microscopic Examination

For microscopic analysis, the filter papers were examined under a NIKON E600 microscope equipped with an Olympus DP75 digital camera (Evident, Tokyo, Japan). The type, color, quantity, and size of the detected microplastics (MPs) were recorded. MPs were first visualized and counted using a 4× objective lens, and detailed images were captured using a 10× objective lens.

Particles of suitable size were marked directly on the Petri dishes and set aside for further analysis using attenuated total reflectance (ATR) and Fourier transform infrared (FTIR).

### 2.4. Fourier Transform Infrared (FTIR) Spectroscopy

Following microscopic examination, Fourier transform infrared (FTIR) spectroscopy was used to determine the chemical composition and origin of the identified microplastics. Since the subsampled amount exceeded 10% of the total isolated microplastics, the polymer distribution of the subsampled MPs represents the entire dataset in accordance with guidelines [57]. This method has been successfully used in studies focusing on microplastic [58,59,60]. FTIR analysis was performed using a PerkinElmer (USA) Spectrum 100 FTIR spectrophotometer equipped with a single-reflection attenuated total reflectance (ATR) accessory.

The spectra were recorded over a range of 4000–600 cm^−1^ with a resolution of 4.0 cm^−1^, and 32 scans were performed for each measurement. Polymer types were identified by comparing the absorbance spectra to reference spectra in the PerkinElmer spectral library.

### 2.5. SEM/EDS Analyses

High-resolution scanning electron microscopy coupled with energy-dispersive X-ray spectroscopy (SEM/EDS) (JEOL, JSM 7001F, Tokyo, Japan) was used to examine the surface morphology and elemental composition of the microplastics. During microscopic analysis, locations with confirmed MP presence were marked, and approximately 0.5 cm × 0.5 cm square fragments were cut from these areas. To ensure conductivity, the samples were coated with a thin layer of gold (Au) prior to SEM observation.

### 2.6. Contamination Prevention

Sampling, digestion, and microscopic examination procedures were conducted in restricted-access, enclosed laboratories to minimize airborne contamination [61]. Throughout these processes, all doors and windows were kept closed [62]. In addition, all operations from digestion to microscopic examination were carried out under a laminar flow cabinet to minimize the risk of airborne contamination. Laboratory surfaces, glass beakers, and all digestion equipment used in the analyses were thoroughly cleaned with ultrapure water before and after each dissection procedure [61].

During all stages of analysis, laboratory personnel wore gloves and cotton lab coats to reduce the risk of contamination. For quality control purposes, two wet blank filters were placed in Petri dishes during digestion and microscopic examination and were inspected under a microscope for the presence of microplastics. No microplastics were detected on the blank filters.

### 2.7. Data Analysis

Microplastic (MP) concentration in fish specimens was reported as the mean number of MPs per individual ± standard deviation (MP/fish). Data normality was evaluated using the Shapiro–Wilk test. Since the normality of length, weight, tract (GIT) weight, and MP data could not be confirmed (*p* < 0.001), Spearman correlation analysis was applied at a 0.05 significance level to investigate the relationship between physical parameters (length, weight, GIT weight) and MP intake rate, as well as to examine potential variations in MP abundance between pelagic and demersal fish.

## 3. Results

In total, 90 fish specimens (*n* = 15 per species) belonging to six different species, both farmed and wild-caught, were collected from the Mid-Black Sea Region (Samsun) and examined for microplastic (MP) abundance in their gastrointestinal tracts. While no microplastics were detected in the control samples, the presence of MPs was observed in all examined individuals from both marine and aquaculture sources.

A total of 639 MPs were isolated from all samples—388 from farmed fish and 251 from wild-caught marine fish. The overall mean MP abundance in the gastrointestinal tracts of all fish was calculated as 7.1 ± 0.05 MPs per individual.

Average MP counts per species were as follows: *Oncorhynchus mykiss* (rainbow trout/Turkish salmon): 9.26 ± 2.18, *Sparus aurata* (gilthead seabream): 9.0 ± 2.05, *Dicentrarchus labrax* (European seabass): 7.6 ± 1.19, *Mullus barbatus* (red mullet): 6.13 ± 1.11, *Trachurus mediterraneus* (horse mackerel): 5.86 ± 0.74, *Merlangius merlangus* (whiting): 4.73 ± 1.13 MPs per fish.

A statistically significant difference in MP abundance was observed between pelagic and demersal fish species (*p* < 0.05), indicating that habitat type may influence microplastic ingestion rates. According to the Spearman analysis performed to determine the differences between species, a strong positive correlation was found between height, weight, and GIT weight *p* ≈ 0.92–0.93), while a low to moderate correlation was found between MP and other variables (*p* ≈ 0.24–0.34) (Supplementary Materials Table S1).

**Figure 1:**
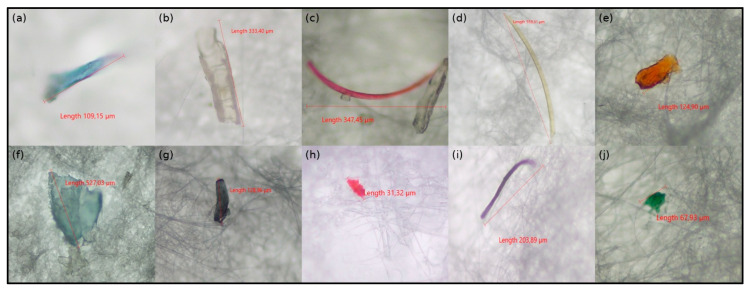
Representative microplastic particles extracted from the gastrointestinal tracts (GITs) of fish samples. (**a**,**f**) blue, (**b**) transparent, (**c**) pink, (**d**) yellow, (**e**) orange, (**g**) black, (**h**) red, (**i**) purple, (**j**) green.

**Figure 2:**
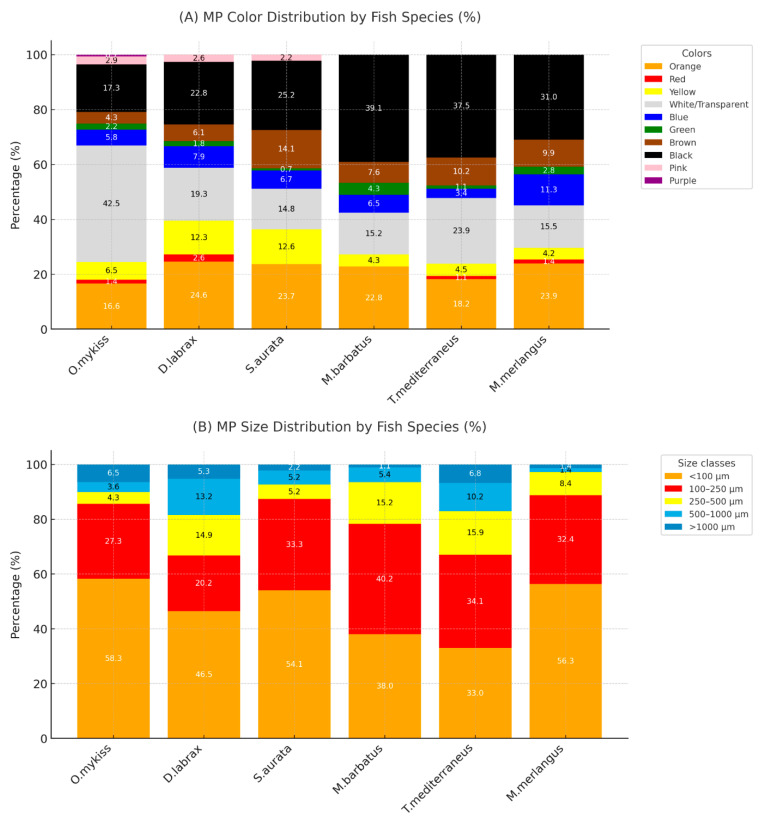
Distribution of microplastic (**A**) colors and (**B**) sizes detected in the analyzed fish species.

Of the 639 isolated microplastics (MPs) particles, 21.6% were orange, 27.5% black, 1.1% red, 8% yellow, 23.1% transparent, 6.8% blue, 2% green, 8.7% brown, 1.6% pink, and 0.2% purple in color (Supplementary Materials Table S2). Figure 1 presents selected examples of microplastics observed in various colors. Figure 2A shows the color distribution percentages of microplastics according to fish species.

In terms of particle size distribution (Figure 2B), the majority of MPs (48.9%) were smaller than 100 µm, followed by 30.8% in the 100–250 µm range, 10% in the 250–500 µm range, 6.6% in the 500–1000 µm range, and 4.1% were larger than 1000 µm (Supplementary Materials Table S3).

The greatest diversity of colored microplastics (MPs) was observed in *O. mykiss*, while *M. barbatus* and *T. mediterraneus* exhibited the lowest diversity. In terms of size distribution, the highest amounts for MP smaller than 100 µm were found in *O. mykiss* (58.3%) and *S. aurata* (54.1%).

**Figure 3:**
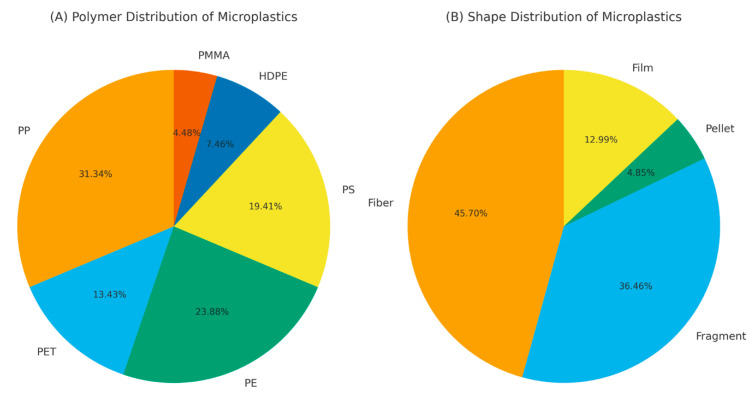
Distribution of (**A**) polymer types and (**B**) microplastic shapes isolated from the gastrointestinal systems of fish sampled in Mid-Black Sea, Samsun.

The MPs identified in the fish samples were classified into four categories: fibers, fragments, pellets, and films (Figure 3B). The proportional distribution of MP shapes was as follows: fibers (45.7%) > fragments (36.4%) > films (13%) > pellets (4.9%).

A total of 67 particles, corresponding to 10.5% of the total microplastics found in the fish, were analyzed by FTIR spectroscopy, and only those with spectra matching the reference data above 70% were classified (Supplementary Materials Figure S1).

According to the FTIR results, among the 67 samples, 31.3% were polypropylene (PP), 23.9% were polyethylene (PE), 19.4% were polystyrene (PS), 13.4% were polyethylene terephthalate (PET), 7.5% were high-density polyethylene (HDPE), and were 4.5% polymethyl methacrylate (PMMA) (Figure 3A).

**Figure 4:**
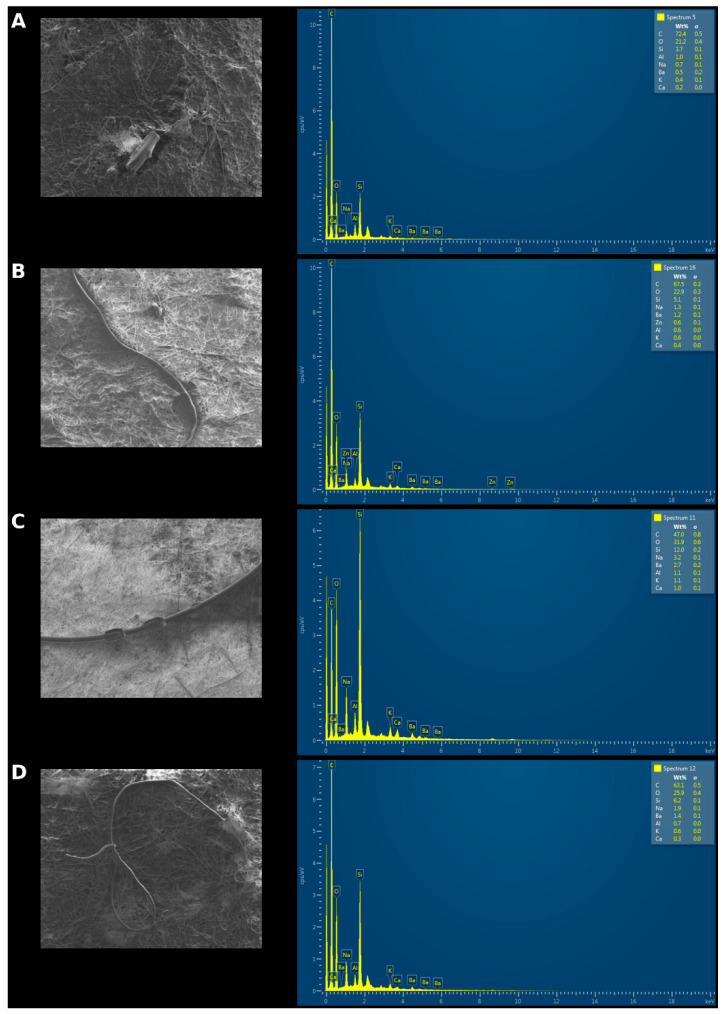
SEM/EDS images of MPs obtained from the GIS of fish samples ((**A**): fragment, (**B**–**D**): Fiber).

The surface morphology of microplastics (MPs) was determined using SEM analysis. According to the images shown in Figure 4, irregular, fibrous, and fragment-like structures typical of microplastic morphology are observed at the microscopic scale. High levels of carbon and oxygen indicate the presence of organic polymer-based materials (such as polyethylene, polypropylene, polystyrene, PET, etc.) The image in Figure 4A contains 72.4% carbon by weight, Figure 4B contains 67.5%, Figure 4C contains 47%, and Figure 4D contains 63.1%. After carbon and oxygen, the presence of silicon (Si) may indicate the presence of fillers in some polymers (e.g., silica) or could be due to environmental contamination.

## 4. Discussion

The rapidly increasing abundance of microplastics in marine and freshwater environments poses a serious threat to ecosystems and aquatic organisms. This situation has led to a significant rise in studies investigating the ingestion behavior of microplastics by aquatic organisms in recent years [37,63]. Research on the interactions between microplastics and aquatic life in marine ecosystems is quite comprehensive [64,65,66]. However, similar knowledge for aquaculture is still limited. In this context, the present study thoroughly examines the presence of microplastics (MP) in six economically important commercial fish species from the Mid-Black Sea Region and reveals the occurrence of MP pollution in each species.

Many studies conducted on commercially consumed fish have found microplastic (MP) occurrence in fish collected from various regions, similar to the present study. These regions include the Adriatic Sea [67], the coastal waters of Mumbai, India [68], and the Shanghai market [69], as well as the northeastern coast of the Arabian Sea [70] and Vellore, Tamil Nadu, India [71], where 100% MP occurrence was observed in the collected fish. Additionally, Kılıç and Yücel [63] reported MP occurrence ranging from 66% to 100% in the İskenderun Bay, Türkiye; ref. [72] found 96% in Tenerife, Canary Islands, Spain; Aytan et al. [73] observed between 10% and 100% in the Eastern Marmara Sea; and Palermo et al. [74] reported 85% in Northern Mindanao, Philippines.

In this study, the average number of microplastics (MPs) ingested by fish (7.1 ± 0.05 MP/fish) is comparable to previous studies conducted in the Black Sea: Barutçu [75] reported 4.27 MP/fish in the Mert River, Samsun; Terzi [76] found 3.2 MP/fish in the Kızılırmak River; Toschkova et al. [77] reported 9.3 MP/fish in the Bulgarian Black Sea; and Ciuca et al. [78] observed 3.22 MP/fish in the Romanian Black Sea. However, some studies from the southern coasts of the Black Sea have reported lower values: Aytan et al. [73] with 0.91 MP/fish; Eryaşar et al. [52] with 0.26 MP/fish; Şentürk et al. [79] with 1.38 MP/fish; Aytan et al. [20] with 0.81 MP/fish; and Tepe et al. [53] with 1.7 MP/fish. In studies conducted in different regions, Aytan et al. [80] reported lower MP abundances than the results of this study, with 1.14 MP/Pisces in the Marmara Sea, Güven et al. [33] with 1.3 MP in the Mediterranean Sea, and Barboza et al. [81] with 0.8 MP/fish in the Northeast Atlantic Ocean. Differences in the abundance of MPs detected in fish samples may depend on factors such as sampling time, sample size, spatial and vertical distribution of MPs in the sampling area, variations in diet types and feeding strategies, behavioral differences among the studied fish species, region-specific plastic pollution, different analytical methods, and sampling protocols [8,11,52,68,69,70].

Microplastic (MP) ingestion by pelagic and demersal fish has been reported worldwide. While some studies found differences between these groups [33,82], others did not [80,83]. In this study, MPs were detected in both groups, but concentrations were higher in demersal species, with significant differences in MP abundance (*p* < 0.05). Demersal fish showed strong positive correlations between length, weight, and GIT weight (r ≈ 0.91–0.94) and a weak to moderate positive correlation with MP abundance (r = 0.21–0.32; *p* < 0.05). Pelagic fish also showed strong correlations among length, weight, and GIT weight (r ≈ 0.96–0.97), but MP abundance was weakly negatively correlated (r ≈ −0.10 to −0.05). Similarly to our findings, several studies reported higher MP loads in demersal fish [62,83,84,85], whereas others found greater ingestion in pelagic species [31,70,85]. The higher MP abundance in demersal species may be related to differences in feeding behaviors and the presence of MPs on the seabed, which is considered the main sink area for MP debris [69,86]. Moreover, it has been reported that demersal species may ingest sediment while feeding on their prey [87,88]. Sediment ingestion behavior is considered one of the pathways through which MPs enter the GIT, and therefore, this may result in higher MP uptake rates in demersal species, consistent with the presence of MPs on the seabed [52,83,89].

Previous studies have shown that the frequency of microplastic (MP) ingestion in fish has increased in parallel with rising plastic pollution in both marine and freshwater ecosystems. For example, Kılıç and Yücel [21] reported higher MP accumulation in fish collected from areas with high plastic pollution in marine environments. Lusher et al. [87] in the English Channel, Foekema et al. [90] in the North Sea, and Collard et al. [91] in the Mediterranean have all indicated a high tendency of MP ingestion in fish. Similarly, Barboza et al. [81] demonstrated the abundance of MPs in fish along the coasts of Portugal, while Arias-Andres et al. [92] confirmed similar trends in freshwater environments.

This study examined the presence of microplastics (MPs) in the gastrointestinal tracts of aquaculture fish species—*O. mykiss*, *D. labrax*, *S. aurata*—and marine species—*M. barbatus*, *T. mediterraneus*, and *M. merlangus*. *O. mykiss* showed the highest MP abundance, averaging 9.26 MP/fish (1.66 MP/g). Comparable results were reported by Alak et al. [93] (1.27 MP/g) and Kılıç [63] (1.2 MP/fish), while Piskula and Astel [94] found lower levels (0.72 MP/fish) in rainbow trout. *S. aurata* followed with 9 MP/fish. Variable results were also reported in previous studies byBessa et al. [95] (3.14 MP), Sánchez-Almeida et al. [50] (5.1 MP/fish), and Eryaşar et al. [85] (0.41 MP/fish). Body size and feeding habits of fish may be factors that facilitate the uptake of microplastics (MPs) from the aquatic environment; therefore, a higher abundance of these particles in the digestive systems of certain species can be expected [94]. Although O. mykiss is raised on farms like other fish, the digestive and absorption surface and permeability status in the intestine may differ from other fish. In vitro studies conducted by Verdile et al. [96] have revealed that epithelial cells in the intestine of *O. mykiss* exhibit specific features in relation to microplastic uptake.

In general, most of these field studies have associated differences in microplastic ingestion with varying feeding strategies of fish [97,98,99], vertical distribution, or location factors such as proximity to urban or industrial areas [69,84,99]. Compared to aquaculture fish, marine-caught fish showed lower MP abundance. Among marine fish, the highest average MP abundance was found in *M. barbatus* (6.13 MP), and the lowest in *M. merlangus* (4.73 MP). In a study conducted by Aytan et al. [100] on three different marine habitats (pelagic, benthopelagic and demersal) in the southeastern Black Sea coast, demersal fish exhibit higher sensitivity to microplastic ingestion compared to pelagic and benthopelagic species. They detected 3.46 MP per individual for *M. barbatus* and 1.28 MP for *M. merlangus*. Onay et al. [37] reported seasonal MP abundance for *M. barbatus* in the southeastern Black Sea, with 1 MP/individual in spring and 4.73 MP/individual in winter. Tepe et al. [53] reported MP presence of 1.9 MP for *M. barbatus* and 1.8 MP for *M. merlangus* along the southeastern Black Sea coasts in Giresun, Türkiye. Differences in sampling and analysis methods, sampling time, sample size, weather conditions, presence and distribution of MPs in the sampling area, and fish behaviors may cause significant variations in the findings of recent studies [52,53].

Microplastics obtained from marine organisms can serve as indicators of plastic pollution in their surrounding environment. Therefore, classification of microplastics in terms of shape, size, and color is crucial for identifying their sources and impacts [101,102]. For example, plastic fibers found in an organism are often linked to clothing fibers discharged into the ocean from wastewater or originate from abandoned fishing lines or nets in the ocean [103,104]. Plastic fragments, on the other hand, correspond to commercial plastics directly dumped into the ocean as a result of anthropogenic activities (such as tourism, fishing, offshore facilities) or poor waste management strategies in some countries [24].

In our study, consistent with all globally compared studies [12,50,53,103,105], the most common type of MP detected was fiber (45.7%), followed by fragments (36.4%). Considering the increasing economic activities along the Black Sea coast and their impact on river water quality [106], river mouths can be regarded as potential sources of MPs. This is particularly associated with the discharge of Türkiye’s two largest rivers, the Kızılırmak and the Yeşilırmak, into the Black Sea.

The current study and the results of previous research agree that polypropylene (PP) and polystyrene (PS) are the most common polymer types [52,94,107]. PP is a type of plastic widely used in clothing, packaging papers, pipes, household, medical, and food industries, textiles, construction, agricultural films, and single-use products [1]. Contrary to our study, similar studies conducted along the Mediterranean and Black Sea coasts [99,108] identified polyethylene terephthalate (PET) as the dominant polymer type. The differences observed in dominant polymer types can be attributed to the variety of industrial activities and consumption habits in the regions where the studies were conducted.

In this study, the most frequently encountered color of MPs obtained from fish was black. Similarly, Gündoğdu et al. [19], Koraltan et al. [109], Kılıç and Yücel [21], and Koongolla et al. [110] reported black as the dominant color. It has been suggested that the main reason benthic fish that mostly feed on sediment consume more black MPs is that these particles resemble their food [111,112]. In contrast, Benaires et al. [4] reported orange as the dominant color in commercial fish in the Philippines, while Cera et al. [113] reported blue as the dominant color in the western Mediterranean Sea, Tyrrhenian region.

## 5. Conclusions

In this study, the abundance and characteristics of microplastics (MPs) were thoroughly examined in six economically valuable fish species (*O. mykiss*, *D. labrax*, *S. aurata*, *M.s barbatus*, *T. mediterraneus*, and *M. merlangus*) along the Samsun–Middle Black Sea. The results revealed the highest MP load in *O. mykiss* (9.26 ± 2.18 MP/individual) and *S. aurata* (9.0 ± 2.05 MP/individual). Regarding particle size, the vast majority of MPs were found to belong to the <100 µm size class (approximately 49%), with fibers (45.7%) and fragments (36.5%) being the dominant morphological types. Color distribution analysis showed that black (27.6%) and white/transparent (23.1%) MPs were more prevalent compared to other color groups. Polymer analysis results indicated that polypropylene (PP, 31.3%) and polyethylene (PE, 23.9%) were the dominant polymers detected in the samples.

These findings suggest that consumption patterns, coastal settlements, and industrial activities in the region are reflected in the composition of microplastic pollution. Therefore, the development of regional strategies prioritizing waste management, recycling practices, and source control to reduce microplastic pollution in the Black Sea ecosystem is of great importance. Additionally, long-term and interdisciplinary monitoring studies considering different seasons, habitats, and trophic levels are expected to contribute to a more comprehensive assessment of the potential impacts of microplastics on fisheries and human health.

## Figures and Tables

**Table 1:** General information on sampled fish species and microplastic (MP) quantities.

Species	*Oncorhynchus mykiss*	*Dicentrarchus labrax*	*Sparus aurata*	*Mullus barbatus*	*Trachurus mediterraneus*	*Merlangius merlangus*
Common name	Rainbow trout	European seabass	Gilthead seabream	Red mullet	Horse mackerel	Whiting
Habitat	Benthopelagic	Demersal	Demersal	Demersal	Pelagic	Benthopelagic
Fish origin	Aquaculture	Aquaculture	Aquaculture	Wild-caught	Wild-caught	Wild-caught
Description	Lives near the bottom and in the water column	Inhabits nearshore, close to the seabed	Lives in rocky and sandy bottom areas	Lives on soft-bottom seabeds	Lives in open water (water column), exhibits migratory behavior	Inhabits the area between the bottom and water column
Sample (*n*)	15	15	15	15	15	15
Total length (cm)	21.74 ± 2.55	23.5 ± 1.98	22.48 ± 2.09	12.98 ± 0.73	13.75 ± 0.70	16.01 ± 0.86
GIT weight (g)	5.56 ± 0.57	6.02 ± 0.93	6.77 ± 0.36	1.81 ± 0.31	1.78 ± 0.27	2.07 ± 0.38
Total weight (g)	132.36 ± 22.64	198.86 ± 31.85	215.64 ± 32.72	23.53 ± 2.05	23.23 ± 0.95	26.80 ± 1.99
Total MP count	139	114	135	92	88	71
MP per fish	9.26 ± 2.18	7.6 ± 1.19	9.0 ± 2.05	6.13 ± 1.11	5.86 ± 0.74	4.73 ± 1.13

## Data Availability

Data will be made available on request.

## Supplementary Materials

The following supporting information can be downloaded at https://www.mdpi.com/article/10.3390/toxics13100865/s1, Figure S1: Sample FTIR spectra of the main identified polymers; Table S1: Spearman correlation matrix of MP abundance values and physical parameters of fish; Table S2: Color distribution of microplastics in fish samples; Table S3: Size distribution of microplastics in fish samples.

## References

[1] Plastic Europe (2023). Plastics—The Fast Facts 2023.

[2] Jambeck J.R., Geyer R., Wilcox C., Siegler T.R., Perryman M., Andrady A., Narayan R., Lavender Law K. (2015). Plastic Waste Input from Land into Ocean. Science.

[3] Hartmann N.B., Hüffer T., Thompson R.C., Hassellöv M., Verschoor A., Daugaard A.E., Rist S., Karlsson T., Brennholt N., Cole M. (2019). Are We Speaking the Same Language? Recommendations for a Definition and Categorization Framework for Plastic Debris. Environ. Sci. Technol..

[4] Benaires K.D., Yap Dejeto L.G., Parilla R.B. (2025). Microplastics in Commercially Sold Fish in a Coastal City of the Philippine Islands, Western Pacific. Reg. Stud. Mar. Sci..

[5] Constant M., Kerhervé P., Mino-Vercellio-Verollet M., Dumontier M., Sànchez Vidal A., Canals M., Heussner S. (2019). Beached Microplastics in the Northwestern Mediterranean Sea. Mar. Pollut. Bull..

[6] Pojar I., Kochleus C., Dierkes G., Ehlers S.M., Reifferscheid G., Stock F. (2021). Quantitative and Qualitative Evaluation of Plastic Particles in Surface Waters of the Western Black Sea. Environ. Pollut..

[7] Gündoğdu S., Çevik C., Güzel E. (2018). Microplastics in Municipal Wastewater Treatment Plants in Turkey: A Comparison of the Influent and Secondary Effluent Concentrations. Environ. Monit. Assess..

[8] Liu R.P., Liu F., Sun P.P., El Wardany R.M., Dong Y., Zhang Y.B., Chen H.Q., Jiao J.G. (2022). Research Progress of Microplastic Pollution in the Vadose Zone. Water.

[9] Zhang J., Ding W., Zou G., Wang X., Zhao M., Guo S., Chen Y. (2023). Urban Pipeline Rainwater Runoff Is an Important Pathway for Land-Based Microplastics Transport to Inland Surface Water: A Case Study in Beijing. Sci. Total Environ..

[10] Anderson J.C., Park B.J., Palace V.P. (2018). Microplastics in Aquatic Environments: Implication for Canadian Environments. Environ. Pollut..

[11] Wang C., O’Connor D., Wang L., Wu W.M., Luo J., Hou D. (2022). Microplastics in Urban Runoff: Global Occurrence and Fate. Water Res..

[12] Bayhan B., Uncumusaoğlu A.A. (2024). Abundance, Characteristics, and Potential Ecological Risks of Microplastics in Some Commercial Fish in İzmir Bay (Aegean Sea, Türkiye). Reg. Stud. Mar. Sci..

[13] Beer S., Garm A., Huwer B., Dierking J., Nielsen T.G. (2018). No Increase in Marine Microplastic Concentration over the Last Three Decades–A Case Study from the Baltic Sea. Sci. Total Environ..

[14] Ji X., Yan S., He Y., He H., Liu H. (2023). Distribution Characteristics of Microplastics in Surface Seawater off the Yangtze River Estuary Section and Analysis of Ecological Risk Assessment. Toxics.

[15] Sun X., Jia Q., Ye J., Zhu Y., Song Z., Guo Y., Chen H. (2023). Real-Time Variabilities in Microplastic Abundance and Characteristics of Urban Surface Runoff and Sewer Overflow in Wet Weather as Impacted by Land Use and Storm Factors. Sci. Total Environ..

[16] Wu F., Wang Y., Leung J.Y., Huang W., Zeng J., Tang Y., Chen J., Shi A., Yu X., Xu X. (2020). Accumulation of Microplastics in Typical Commercial Aquatic Species: A Case Study at a Productive Aquaculture Site in China. Sci. Total Environ..

[17] Yozukmaz A. (2021). Investigation of Microplastics in Edible Wild Mussels from İzmir Bay (Aegean Sea, Western Turkey): A Risk Assessment for the Consumers. Mar. Pollut. Bull..

[18] Abbasi S., Soltani N., Keshavarzi B., Moore F., Turner A., Hassanaghaei M. (2018). Microplastics in Different Tissues of Fish and Prawn from the Musa Estuary, Persian Gulf. Chemosphere.

[19] Gündogdu S., Cevik C., Atas¸ N.T. (2020). Occurrence of Microplastics in the Gastrointestinal Tracts of Some Edible Fish Species along the Turkish Coast. Turk. J. Zool..

[20] Aytan U., Esensoy F.B., Senturk Y., Arifoglu E., Karaoglu K., Ceylan Y., Valente A. (2021). Plastic Occurrence in Commercial Fish Species of the Black Sea. Turk. J. Fish. Aquat. Sci..

[21] Kılıç E., Yücel N. (2022). Microplastic Occurrence in the Gastrointestinal Tract and Gill of Bioindicator Fish Species in the Northeastern Mediterranean. Mar. Pollut. Bull..

[22] Walkinshaw C., Lindeque P.K., Thompson R., Tolhurst T., Cole M. (2020). Microplastics and Seafood: Lower Trophic Organisms at Highest Risk of Contamination. Ecotoxicol. Environ. Saf..

[23] Ivleva N.P., Wiesheu A.C., Niessner R. (2017). Microplastic in Aquatic Ecosystems. Angew. Chem. Int. Ed..

[24] Rochman C.M., Tahir A., Williams S.L., Baxa D.V., Lam R., Miller J.T., Teh F.-C., Werorilangi S., Teh S.J. (2015). Anthropogenic Debris in Seafood: Plastic Debris and Fibers from Textiles in Fish and Bivalves Sold for Human Consumption. Sci. Rep..

[25] Tien C.J., Wang Z.X., Chen C.S. (2020). Microplastics in Water, Sediment and Fish from the Fengshan River System: Relationship to Aquatic Factors and Accumulation of Polycyclic Aromatic Hydrocarbons by Fish. Environ. Pollut..

[26] Koelmans A.A., Redondo-Hasselerharm P.E., Nor N.H.M., de Ruijter V.N., Mintenig S.M., Kooi M. (2022). Risk Assessment of Microplastic Particles. Nat. Rev. Mater..

[27] Wright S.L., Kelly F.J. (2017). Plastic and Human Health: A Micro Issue?. Environ. Sci. Technol..

[28] Silva J.D.S., Rodrigues J.R.P., Sá R.A.D.Q.C.D., de Oliveira M.B.M., da Silva S.M., Souza K.S., da Silva M.R.F., de Araújo L.C.A., Pereira E.S., Bezerra A.Â. (2022). Environmental Pollution by Microplastics and Its Consequences on Human Health. Res. Soc. Dev..

[29] Feng Z., Zhang T., Wang J., Huang W., Wang R., Xu J., Fu G., Gao G. (2020). Spatiotemporal Features of Microplastics Pollution in Macroalgae Growing in an Important Mariculture Area, China. Sci. Total Environ..

[30] Gao G., Zhao X., Jin P., Gao K., Beardall J. (2021). Current Understanding and Challenges for Aquatic Primary Producers in a World with Rising Micro- and Nanoplastic Levels. J. Hazard. Mater..

[31] Kılıç E., Yücel N., Sahutoğlu S.M. (2022). First Record of Microplastic Occurrence at the Commercial Fish from Orontes River. Environ. Pollut..

[32] Fossi M.C., Panti C., Baini M., Lavers J.L. (2018). A Review of Plastic Associated Pressures: Cetaceans of the Mediterranean Sea and Eastern Australian Shearwaters as Case Studies. Front. Mar. Sci..

[33] Güven O., Gokdağ K., Jovanović B., Kıdeyş A.E. (2017). Microplastic Litter Composition of the Turkish Territorial Waters of the Mediterranean Sea, and Its Occurrence in the Gastrointestinal Tract of Fish. Environ. Pollut..

[34] Jovanovic B. (2017). Ingestion of Microplastics by Fish and Its Potential Consequences from a Physical Perspective. Integr. Environ. Assess. Manag..

[35] Miller M.E., Hamann M., Kroon F.J. (2020). Bioaccumulation and Biomagnification of Microplastics in Marine Organisms: A Review and Meta-Analysis of Current Data. PLoS ONE.

[36] McIlwraith H.K., Kim J., Helm P., Bhavsar S.P., Metzger J.S., Rochman C.M. (2021). Evidence of Microplastic Translocation in Wild-Caught Fish and Implications for Microplastic Accumulation Dynamics in Food Webs. Environ. Sci. Technol..

[37] Onay H., Minaz M., Ak K., Er A., Emanet M., Karslı B., Bilgin S. (2023). Decade of Microplastic Alteration in the Southeastern Black Sea: An Example of Seahorse Gastrointestinal Tracts. Environ. Res..

[38] Kalipci E., Cuce H., Temel F.A., Dereli M.A., Turkmen A. (2024). Microplastic Pollution Profile in the Black Sea Region. Curr. Innov. Chem. Mater. Sci..

[39] Bhuyan M.S. (2022). Effects of Microplastics on Fish and in Human Health. Front. Environ. Sci..

[40] Kılıç E. (2024). Abundance and Ecological Risk of Microplastics in Commercial Fish Species from Northeastern Mediterranean Sea. Environ. Pollut..

[41] Sequeira I.F., Prata J.C., da Costa J.P., Duarte A.C., Rocha-Santos T. (2020). Worldwide Contamination of Fish with Microplastics: A Brief Global Overview. Mar. Pollut. Bull..

[42] Hantoro I., Löhr A.J., Van Belleghem F.G.A.J., Widianarko B., Ragas A.M.J. (2019). Microplastics in Coastal Areas and Seafood: Implications for Food Safety. Food Addit. Contam., Part A.

[43] Hermabessiere L., Dehaut A., Paul-Pont I., Lacroix C., Jézéquel R., Soudant P., Duflos G. (2017). Occurrence and Effects of Plastic Additives on Marine Environments and Organisms: A Review. Chemosphere.

[44] Cabansag J.B.P., Olimberio R.B., Villanobos Z.M.T. (2021). Microplastics in Some Fish Species and Their Environs in Eastern Visayas, Philippines. Mar. Pollut. Bull..

[45] Barboza L.G.A., Vieira L.R., Guilhermino L. (2018). Single and Combined Effects of Microplastics and Mercury on Juveniles of the European Seabass (*Dicentrarchus labrax*): Changes in Behavioural Responses and Reduction of Swimming Velocity and Resistance Time. Environ. Pollut..

[46] Santana M.F., Dawson A.L., Motti C.A., van Herwerden L., Lefevre C., Kroon F.J. (2021). Ingestion and Depuration of MPs by a Planktivorous Coral Reef Fish, *Pomacentrus amboinensis*. Front. Environ. Sci..

[47] Alprol A.E., Gaballah M.S., Hassaan M.M. (2021). Micro and Nanoplastics Analysis: Focus on Their Classification, Sources, and Impacts in Marine Environment. Reg. Stud. Mar. Sci..

[48] Carbery M., O’Connor W., Palanisami T. (2018). Trophic Transfer of Microplastics and Mixed Contaminants in the Marine Food Web and Implications for Human Health. Environ. Int..

[49] Turkish Statistical Institute (TUIK) (2024). Aquaculture Statistics. https://data.tuik.gov.tr/Bulten/Index?p=Su-Urunleri-2023-53702.

[50] Sánchez-Almeida R., Hernández-Sánchez C., Villanova-Solano C., Díaz-Peña F.J., Clemente S., González-Sálamo J., González-Pleiter M., Hernández-Borges J. (2022). Microplastics Determination in Gastrointestinal Tracts of European Sea Bass (*Dicentrarchus labrax*) and Gilt-Head Sea Bream (*Sparus aurata*) from Tenerife (Canary Islands, Spain). Polymers.

[51] Gedik K. (2018). Bioaccessibility of Cd, Cr, Cu, Mn, Ni, Pb, and Zn in Mediterranean Mussel (*Mytilus galloprovincialis* Lamarck, 1819) along the Southeastern Black Sea Coast. Hum. Ecol. Risk Assess..

[52] Eryasar A.R., Gedik K., Mutlu T. (2022). Ingestion of Microplastics by Commercial Fish Species from the Southern Black Sea Coast. Mar. Pollut. Bull..

[53] Tepe Y., Aydın H., Ustaoğlu F., Kodat M. (2024). Occurrence of Microplastics in the Gastrointestinal Tracts of Four Most Consumed Fish Species in Giresun, the Southeastern Black Sea. Environ. Sci. Pollut. Res..

[54] Perumal K., Muthuramalingam S. (2021). Global Sources, Abundance, Size, and Distribution of Microplastics in Marine Sediments—A Critical Review. Estuar. Coast. Shelf Sci..

[55] Avio C.G., Gorbi S., Regoli F. (2015). Experimental Development of a New Protocol for Extraction and Characterization of Microplastics in Fish Tissues: First Observations in Commercial Species from Adriatic Sea. Mar. Environ. Res..

[56] Renzi M., Specchiulli A., Blasković A., Manzo C., Mancinelli G., Cilenti L. (2019). Marine Litter in Stomach Content of Small Pelagic Fishes from the Adriatic Sea: Sardines (*Sardina pilchardus*) and Anchovies (*Engraulis encrasicolus*). Environ. Sci. Pollut. Res..

[57] Hanke G., Galgani F., Werner S., Oosterbaan L., Nilsson P., Fleet D., Kinsey S., Thompson R.C., Frakener J.V., Sco-ullos M. (2013). Guidance on Monitoring of Marine Litter in European Seas. Scientific and Technical Research Series, EUR 26113 EN. https://mcc.jrc.ec.europa.eu/documents/201702074014.pdf.

[58] Digka N., Tsangaris C., Torre M., Anastasopoulou A., Zeri C. (2018). Microplastics in Mussels and Fish from the Northern Ionian Sea. Mar. Pollut. Bull..

[59] Tsangaris C., Digka N., Valente T., Aguilar A., Borrell A., De Lucia G.A., Gambaiani D., Garcia-Garin O., Kaberi H., Martin J. (2020). Using Boops boops (Osteichthyes) to Assess Microplastic Ingestion in the Mediterranean Sea. Mar. Pollut. Bull..

[60] Mistri M., Sfriso A.A., Casoni E., Nicoli M., Vaccaro C., Munari C. (2022). Microplastic Accumulation in Commercial Fish from the Adriatic Sea. Mar. Pollut. Bull..

[61] Bessa F., Frias J., Kögel T., Lusher A., Andrade J.M., Antunes J., Gerdts G. (2019). Harmonized Protocol for Monitoring Microplastics in Biota.

[62] Torre M., Digka N., Anastasopoulou A., Tsangaris C., Mytilineou C. (2016). Anthropogenic Microfibres Pollution in Marine Biota: A New and Simple Methodology to Minimize Airborne Contamination. Mar. Pollut. Bull..

[63] Kılıç E. (2022). Microplastic Ingestion Evidence by Economically Important Farmed Fish Species from Turkey. Mar. Pollut. Bull..

[64] Deudero S., Alomar C. (2015). Mediterranean Marine Biodiversity under Threat: Reviewing Influence of Marine Litter on Species. Mar. Pollut. Bull..

[65] Yu R.S., Singh S. (2023). Microplastic Pollution: Threats and Impacts on Global Marine Ecosystems. Sustainability.

[66] Pal D., Prabhakar R., Barua V.B., Zekker I., Burlakovs J., Krauklis A., Vincevica-Gaile Z. (2025). Microplastics in Aquatic Systems: A Comprehensive Review of Its Distribution, Environmental Interactions, and Health Risks. Environ. Sci. Pollut. Res..

[67] Anastasopoulou A., Viršek M.K., Varezić D.B., Digka N., Fortibuoni T., Koren Š., Tutman P. (2018). Assessment on Marine Litter Ingested by Fish in the Adriatic and NE Ionian Sea Macro-Region (Mediterranean). Mar. Pollut. Bull..

[68] Debbarma N., Gurjar U.R., Ramteke K.K., Shenoy L., Nayak B.B., Bhushan S., Xavier M. (2022). Abundance and Characteristics of Microplastics in Gastrointestinal Tracts and Gills of Croaker Fish (*Johnius dussumieri*) from off Mumbai Coastal Waters of India. Mar. Pollut. Bull..

[69] Jabeen K., Su L., Li J., Yang D., Tong C., Mu J., Shi H. (2017). Microplastics and Mesoplastics in Fish from Coastal and Fresh Waters of China. Environ. Pollut..

[70] Gurjar U.R., Xavier K.M., Shukla S.P., Deshmukhe G., Jaiswar A.K., Nayak B.B. (2021). Incidence of Microplastics in Gastrointestinal Tract of Golden Anchovy (*Coilia dussumieri*) from North East Coast of Arabian Sea: The Ecological Perspective. Mar. Pollut. Bull..

[71] Mohan A.V., Kuttykattil A., Toshiaki I., Sudhakaran R. (2024). Assessment of Microplastic Contamination in Commercially Available Fishes. Mar. Environ. Res..

[72] Reinold S., Herrera A., Saliu F., Hernández-González C., Martinez I., Lasagni M., Gómez M. (2021). Evidence of Microplastic Ingestion by Cultured European Sea Bass (*Dicentrarchus labrax*). Mar. Pollut. Bull..

[73] Aytan U., Senturk Y., Esensoy F.B., Oztekin A., Agırbas E., Valente A. (2020). Microplastic Pollution along the Southeastern Black Sea. Mar. Litter Black Sea.

[74] Palermo J.D., Labrador K.L., Follante J.D., Agmata A.B., Pante M.J., Rollon R.N., David L.T. (2020). Susceptibility of *Sardinella lemuru* to Emerging Marine Microplastic Pollution. Glob. J. Environ. Sci. Manag..

[75] Barutçu E.Ç. (2022). Detection of Microplastics in the Digestive Systems of Some Fish Species Living in the Mert River (Samsun). Master’s Thesis.

[76] Terzi Y. (2023). Microplastic Ingestion by Invasive Prussian Carp (*Carassius gibelio*) Used in Fishmeal Production in Türkiye. Environ. Monit. Assess..

[77] Toschkova S., Ibryamova S., Bachvarova D.C., Koynova T., Stanachkova E., Ivanov R., Ignatova-Ivanova T. (2024). The Assessment of the Bioaccumulation of Microplastics in Key Fish Species from the Bulgarian Aquatory of the Black Sea. BioRisk.

[78] Ciucă A.-M., Stoica E., Barbeș L. (2025). First Report of Microplastic Ingestion and Bioaccumulation in Commercially Valuable European Anchovies (*Engraulis encrasicolus*, Linnaeus, 1758) from the Romanian Black Sea Coast. J. Mar. Sci. Eng..

[79] Şentürk Y., Emanet M., Ceylan Y., Aytan U. (2023). The First Evidence of Microplastics Occurrence in Greater Pipefish (*Syngnathus acus* Linnaeus, 1758) in the Black Sea. Turk. J. Fish. Aquat. Sci..

[80] Aytan Ü., Esensoy F.B., Şentürk Y., Güven O., Karaoğlu K., Erbay M. (2023). Plastic Occurrence in Fish Caught in the Highly Industrialized Gulf of İzmit (Eastern Sea of Marmara, Türkiye). Chemosphere.

[81] Barboza L.G., Vethaak A.D., Lavorante B.R., Lundebye A.K., Guilhermino L. (2018). Marine Microplastic Debris: An Emerging Issue for Food Security, Food Safety and Human Health. Mar. Pollut. Bull..

[82] Sparks C., Immelman S. (2020). Microplastics in Offshore Fish from the Agulhas Bank, South Africa. Mar. Pollut. Bull..

[83] Neves D., Sobral P., Ferreira J.L., Pereira T. (2015). Ingestion of Microplastics by Commercial Fish off the Portuguese Coast. Mar. Pollut. Bull..

[84] Bellas J., Martinez-Armental J., Martinez-Camara A., Besada V., Martinez-Gomez C. (2016). Ingestion of Microplastics by Demersal Fish from the Spanish Atlantic and Mediterranean Coasts. Mar. Pollut. Bull..

[85] Eryaşar A.R., Mutlu T., Karaoğlu K., Veske E., Gedik K. (2024). Assessment of Microplastic Pollution in Eleven Commercial Fish Species in the Gulf of İzmir (Aegean Sea, Eastern Mediterranean). Mar. Pollut. Bull..

[86] Wootton N., Reis-Santos P., Gillanders B.M. (2021). Microplastic in Fish—A Global Synthesis. Rev. Fish Biol. Fish..

[87] Lusher A.L., McHugh M., Thompson R.C. (2013). Occurrence of Microplastics in the Gastrointestinal Tract of Pelagic and Demersal Fish from the English Channel. Mar. Pollut. Bull..

[88] Kühn S., Bravo Rebolledo E.L., van Franeker J.A., Bergmann M., Gutow L., Klages M. (2015). Deleterious Effects of Litter on Marine Life. Marine Anthropogenic Litter.

[89] McGoran A.R., Cowie P.R., Clark P.F., McEvoy J.P., Morritt D. (2018). Ingestion of Plastic by Fish: A Comparison of Thames Estuary and Firth of Clyde Populations. Mar. Pollut. Bull..

[90] Foekema E.M., Gruijter C.D., Mergia M.T., van Franeker J.A., Murk A.J., Koelmans A.A. (2013). Plastic in North Sea Fish. Environ. Sci. Technol..

[91] Collard F., Gilbert B., Compère P., Eppe G., Das K., Jauniaux T., Parmentier E. (2017). Microplastics in Livers of European Anchovies (*Engraulis encrasicolus*, L.). Environ. Pollut..

[92] Arias-Andres M., Kettner M.T., Miki T., Grossart H.P. (2018). Microplastics: New Substrates for Heterotrophic Activity Contribute to Altering Organic Matter Cycles in Aquatic Ecosystems. Sci. Total Environ..

[93] Alak G., Köktürk M., Ucar A., Parlak V., Kocaman E.M., Atamanalp M. (2022). Thermal Processing Implications on Microplastics in Rainbow Trout Fillet. J. Food Sci..

[94] Piskuła P., Astel A. (2024). Occurrence of Microplastics in Commercial Fishes from Aquatic Ecosystems of Northern Poland. Ecohydrol. Hydrobiol..

[95] Bessa F., Barria P., Neto J.M., Frias J.P.G.L., Otero V., Sobral P., Marques J.C. (2018). Occurrence of Microplastics in Commercial Fish from a Natural Estuarine Environment. Mar. Pollut. Bull..

[96] Verdile N., Cattaneo N., Camin F., Zarantoniello M., Conti F., Cardinaletti G., Brevini T.A.L., Olivotto I., Gandolfi F. (2025). New Insights in Microplastic Cellular Uptake Through a Cell-Based Organotypic Rainbow-Trout (*Oncorhynchus mykiss*) Intestinal Platform. Cells.

[97] Romeo T., Pietro B., Peda C., Consoli P., Andaloro F., Fossi M.C. (2015). First Evidence of Presence of Plastic Debris in Stomach of Large Pelagic Fish in the Mediterranean Sea. Mar. Pollut. Bull..

[98] Peters C.A., Thomas P.A., Rieper K.B., Bratton S.P. (2017). Foraging Preferences Influence Microplastic Ingestion by Six Marine Fish Species from the Texas Gulf Coast. Mar. Pollut. Bull..

[99] Silva-Cavalcanti J.S., Silva J.D.B., de França E.J., de Araújo M.C.B., Gusmão F. (2017). Microplastics Ingestion by a Common Tropical Freshwater Fishing Resource. Environ. Pollut..

[100] Aytan Ü., Koca Y.Ş., Pasli S., Güven O., Ceylan Y., Basaran B. (2025). Microplastics in Commercial Fish and Their Habitats in the Important Fishing Ground of the Black Sea: Characteristic, Concentration, and Risk Assessment. Mar. Pollut. Bull..

[101] Wieczorek A.M., Morrison L., Croot P.L., Allcock A.L., MacLoughlin E., Savard O., Brownlow H., Doyle T.K. (2018). Frequency of Microplastics in Mesopelagic Fishes from the Northwest Atlantic. Front. Mar. Sci..

[102] Jaafar N., Azfaralariff A., Musa S.M., Mohamed M., Yusoff A.H., Lazim A.M. (2021). Occurrence, Distribution and Characteristics of Microplastics in Gastrointestinal Tract and Gills of Commercial Marine Fish from Malaysia. Sci. Total Environ..

[103] Hidalgo-Ruz V., Gutow L., Thompson R.C., Thiel M. (2012). Microplastics in the Marine Environment: A Review of the Methods Used for Identification and Quantification. Environ. Sci. Technol..

[104] Khalik W.M.A.W.M., Ibrahim Y.S., Anuar S.T., Govindasamy S., Baharuddin N.F. (2018). Microplastics Analysis in Malaysian Marine Waters: A Field Study of Kuala Nerus and Kuantan. Mar. Pollut. Bull..

[105] Sánchez-Guerrero-Hernández M.J., González-Fernández D., Sendra M., Ramos F., Yeste M.P., González-Ortegón E. (2023). Contamination from Microplastics and Other Anthropogenic Particles in the Digestive Tracts of the Commercial Species *Engraulis encrasicolus* and *Sardina pilchardus*. Sci. Total Environ..

[106] Gartsiyanova K., Genchev S., Kitev A. (2024). Assessment of Water Quality as a Key Component in the Water–Energy–Food Nexus. Hydrology.

[107] Cocci P., Gabrielli S., Pastore G., Minicucci M., Mosconi G., Palermo F.A. (2022). Microplastics Accumulation in Gastrointestinal Tracts of *Mullus barbatus* and *Merluccius merluccius* Is Associated with Increased Cytokine Production and Signaling. Chemosphere.

[108] Compa M., Alomar C., Wilcox C., van Sebille E., Lebreton L., Hardesty B.D., Deudero S. (2019). Risk Assessment of Plastic Pollution on Marine Diversity in the Mediterranean Sea. Sci. Total Environ..

[109] Koraltan İ., Mavruk S., Güven O. (2022). Effect of Biological and Environmental Factors on Microplastic Ingestion of Commercial Fish Species. Chemosphere.

[110] Koongolla J.B., Lin L., Yang C.-P., Pan Y.-F., Li H.-X., Liu S., Xu X.-R. (2022). Microplastic Prevalence in Marine Fish from Onshore Beibu Gulf, South China Sea. Front. Mar. Sci..

[111] Ferreira M., Thompson J., Paris A., Rohindra D., Rico C. (2020). Presence of Microplastics in Water, Sediments and Fish Species in an Urban Coastal Environment of Fiji, a Pacific Small Island Developing State. Mar. Pollut. Bull..

[112] Atamanalp M., Köktürk M., Uçar A., Duyar H.A., Özdemir S., Parlak V., Esenbuğa N., Alak G. (2021). Microplastics in Tissues (Brain, Gill, Muscle and Gastrointestinal) of *Mullus barbatus* and *Alosa immaculata*. Arch. Environ. Contam. Toxicol..

[113] Cera A., Secco S., Matarazzi I., Orsini M., De Santis S., Scalici M. (2025). Ingestion of Prey Intensifies Microplastic Load in Mediterranean Commercial Fish. Cont. Shelf Res..

