# Supplementary material for: Determination of Microplastic Pollution in Commercial Fish in the Middle Black Sea (Samsun), Türkiye
Source: Toxics. 2025 Oct 12;13(10):865. doi: 10.3390/toxics13100865 (PMC12567620; doi:10.3390/toxics13100865)
Supplement: Supplementary file 1 [file toxics-13-00865-s001.zip › toxics-3906761-supplementary.pdf]

Supplemental Material

Determination of Microplastic Pollution in Commercial Fish in the Middle Black Sea (Samsun), Türkiye

Arife Şimşek

Hemp Research Institute, Ondokuz Mayıs University, Samsun 55139, Türkiye; arife.simsek@omu.edu.tr

CONTENTS

Table S1

Table S2

Table S3

Figure S1

Table S1. Spearman correlation matrix of MP abundance values and physical parameters of fish.

	**Length (cm)**	**Weight (gr)**	**MP**	**GIT (gr)**
**Length (cm)**	1.00	**0.93**	0.24	**0.92**
**Weight (cm)**	**0.93**	1.00	0.34	**0.93**
**MP**	0.24	0.34	1.00	0.29
**GIT (gr)**	**0.92**	**0.93**	0.29	1.00

Table S2. Color distribution of microplastics in fish samples.

	Colors									
	Orange	Red	Yellov	White/Transparent	Blue	Green	Brown	Black	Pink	Purple
O.mykiss	23	2	9	59	8	3	6	24	4	1
D. labrax	28	3	14	22	9	2	7	26	3	0
S.aurata	32	0	17	20	9	1	19	34	3	0
M.barbatus	21	0	4	14	6	4	7	36	0	0
T. mediterraneus	16	1	4	21	3	1	9	33	0	0
M. merlangus	17	1	3	11	8	2	7	22	0	0
Total	137	7	51	147	43	13	55	175	10	1
%	21.6	1.1	8.0	23.1	6.8	2.0	8.7	27.5	1.6	0.2

Table S3. Size distribution of microplastics in fish samples.

	MP dimensions				
	<100 μm	100-250 μm	250-500 μm	500-1000 μm	>1000 μm
O.mykiss	81	38	6	5	9
D. labrax	53	23	17	15	6
S.aurata	73	45	7	7	3
M.barbatus	35	37	14	5	1
T. mediterraneus	29	30	14	9	6
M. merlangus	40	23	6	1	1
Total	311	196	64	42	26
%	48.9	30.8	10.0	6.6	4.1

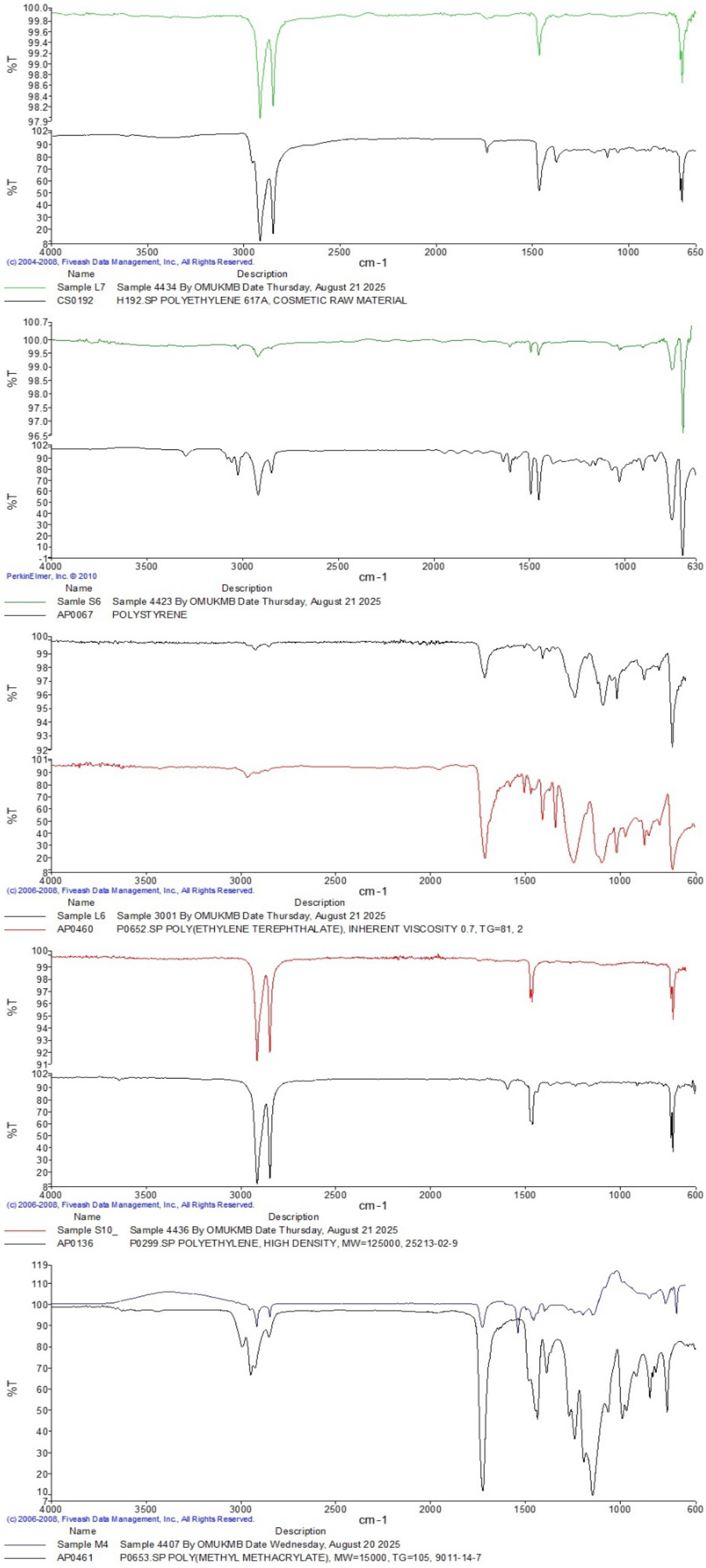

Figure S1. Sample FTIR spectra of the main identified polymers.
